# Prediction of outcome of early ER+ breast cancer is improved using a biomarker panel, which includes Ki-67 and p53

**DOI:** 10.1038/bjc.2011.228

**Published:** 2011-06-28

**Authors:** E K A Millar, P H Graham, C M McNeil, L Browne, S A O'Toole, A Boulghourjian, J H Kearsley, G Papadatos, G Delaney, C Fox, E Nasser, A Capp, R L Sutherland

**Affiliations:** 1 Cancer Research Program, Garvan Institute of Medical Research, 384 Victoria Street, Darlinghurst, New South Wales 2010, Australia; 2 Department of Anatomical Pathology, South Eastern Area Laboratory Service, St George Hospital Kogarah, New South Wales 2217, Australia; 3 School of Medicine and Health Sciences, University of Western Sydney, Campbelltown, New South Wales, Australia; 4 Faculty of Medicine, University of NSW, Kensington, New South Wales, Australia; 5 Department of Radiation Oncology, Cancer Care Centre, St George Hospital Kogarah, New South Wales 2217, Australia; 6 Department of Medical Oncology, Royal Prince Alfred Hospital, University of Sydney, Camperdown, New South Wales, Australia; 7 Department of Diagnostic Oncology and Tissue Pathology, Royal Prince Alfred Hospital, Camperdown, New South Wales 2010, Australia; 8 University of Sydney, Camperdown, New South Wales, Australia; 9 Macarthur Cancer Therapy Centre, Campbelltown, New South Wales, Australia; 10 Department of Radiation Oncology, Liverpool Hospital, Liverpool, UK; 11 Department of Radiation Oncology, Wollongong Hospital, Wollongong, New South Wales, Australia; 12 Department of Radiation Oncology, Mater Misericordiae Hospital, Waratah, New South Wales, Australia; 13 St Vincent's Clinical School, Faculty of Medicine, University of NSW, Darlinghurst, New South Wales 2052, Australia

**Keywords:** breast cancer, biomarker, Ki67, p53, luminal B

## Abstract

**Background:**

The aim of this study is to determine whether immunohistochemical (IHC) assessment of Ki67 and p53 improves prognostication of oestrogen receptor-positive (ER+) breast cancer after breast-conserving therapy (BCT). In all, 498 patients with invasive breast cancer from a randomised trial of BCT with or without tumour bed radiation boost were assessed using IHC.

**Methods:**

The ER+ tumours were classified as ‘luminal A’ (LA): ER+ and/or PR+, Ki-67 low, p53−, HER2− or ‘luminal B’ (LB): ER+ and/or PR+and/or Ki-67 high and/or p53+ and/or HER2+. Kaplan–Meier and Cox proportional hazards methodology were used to ascertain relationships to ispilateral breast tumour recurrence (IBTR), locoregional recurrence (LRR), distant metastasis-free survival (DMFS) and breast cancer-specific survival (BCSS).

**Results:**

In all, 73 patients previously LA were re-classified as LB: a greater than four-fold increase (4.6–19.3%) compared with ER, PR, HER2 alone. In multivariate analysis, the LB signature independently predicted LRR (hazard ratio (HR) 3.612, 95% CI 1.555–8.340, *P*=0.003), DMFS (HR 3.023, 95% CI 1.501–6.087, *P*=0.002) and BCSS (HR 3.617, 95% CI 1.629–8.031, *P*=0.002) but not IBTR.

**Conclusion:**

The prognostic evaluation of ER+ breast cancer is improved using a marker panel, which includes Ki-67 and p53. This may help better define a group of poor prognosis ER+ patients with a greater probability of failure with endocrine therapy.

Oestrogen receptor-positive (ER+) breast cancer comprises approximately 75% of all breast cancers and treatments targeting oestrogen synthesis (aromatase inhibitors) or the ER (tamoxifen) are the most effective adjuvant therapies. Gene expression profiling (GEP) studies over the past decade have established molecular subtypes of ER+ luminal disease, which are characterised by differences in outcome and underlying biology, largely now referred to as luminal A (LA) or luminal B (LB), the latter being characterised by increased proliferation and higher grade as well as lower levels of ER related genes (Perou *et al*, 2000; Sørlie *et al*, 2001). Despite the successes of endocrine therapy in reducing annual recurrences and death by 41% and 34%, respectively, resistance occurs in about 30% of patients treated with tamoxifen (Early Breast Cancer Trialists’ Collaborative Group (EBCTCG), 2005). Therefore, predicting the likely prognosis in an individual patient before treatment would allow early selection of optimal therapies, the importance of which is highlighted in the most recent St Gallen guidelines for the treatment of early breast cancer (Goldhirsch *et al*, 2009).

The abundant data derived from GEP studies have clearly identified the significance of genomic grade and proliferation signatures in prognosis and response to endocrine therapy (reviewed in detail in Musgrove and Sutherland, 2009 and Sotiriou and Pusztai, 2009). However, given the current costs of such molecular testing, translating these findings into an economical, reproducible and readily applicable panel for immunohistochemistry (IHC) in a routine pathology setting is a priority. Most previous IHC definitions of LA and LB tumours include ER+ and/or PR+, with HER2 positivity defining LB, creating a population size of approximately 5–10% (Cheang *et al*, 2008; Nguyen *et al*, 2008; Millar *et al*, 2009b; Blows *et al*, 2010). However, GEP studies have documented the LB population to be larger than this, averaging approximately 16% (ranging from 10 to 21%, reviewed in detail in Sorlie *et al*, 2003 and Hu *et al*, 2006), suggesting that this poorer prognosis subtype may be under-represented using this definition. This discrepancy is most likely explained by the fact that only approximately 30% of LB cancers are in fact HER2 positive (Carey *et al*, 2006). Although proliferation is the key discriminator of luminal tumours, the optimal subclassification of luminal tumours by GEP has yet to be defined (Weigelt *et al*, 2010b). Several studies have, however, shown that intrinsic subtype as defined by IHC ‘mirrors’ the subtypes identified by GEP and that the IHC subtypes so defined have distinct clinical outcomes (Neilsen *et al*, 2004; Abd El-Rehim *et al*, 2005; Cheang *et al*, 2008, 2009; Blows *et al*, 2010). Such IHC definitions are now in common clinical usage. Some recent studies have addressed the issue of a more refined definition of good and poor prognosis ER+ cancer, and used a modified IHC definition to include assessment of the proliferation marker Ki-67 (Cheang *et al*, 2009; Cuzick *et al*, 2009; Hugh *et al*, 2009), which results in a larger proportion of LB tumours with independent prognostic power (Cheang *et al*, 2009). This latter study defined a Ki67 cutpoint (14%) derived from GEP analyses. This set of biomarkers more closely resembles the Oncotype Dx assay of known predictive and prognostic power in ER+, lymph node-negative cancer, which is largely driven by proliferation, HER2- and ER-related genes (Paik *et al*, 2004). However, a recent head to head comparison of a four IHC biomarker panel of ER, PR, HER2 and Ki-67 (IHC 4) has been shown to provide prognostic information, which is at least equivalent to Oncotype Dx using material from the ATAC trial (Cuzick *et al*, 2009). This important study identifies the robustness of prognostic data, which can be provided by routine IHC. Some observers support the view that GEP currently offers no more that routine IHC when combined with important morphological features (not assessable by GEP), such as lymphatic vascular invasion and lymph node status (Weigelt and Reis-Filho, 2010). In addition, these routine analyses can be performed at a fraction of the cost of commercially available GEP tests. In addition, it also supports the concept that measurement of a few well chosen protein products can identify clinically significant patient groups (Ring *et al*, 2006). Histological grade is a key component of routine pathology reporting and of prognostic importance, but may, in some circumstances, be affected by subjectivity, along with problems with inadequate or delayed fixation, which can result in undergrading (Rakha *et al*, 2010). Incorporation of biomarkers as surrogates for molecular grade into routine reporting may help more reliably define good and poor prognosis patients, most significantly for grade 2 invasive carcinomas, which comprise 37–49% of all breast cancers (Rakha *et al*, 2010).

To further validate an IHC panel of markers for routine application in a clinical setting, we assessed a new biomarker panel to differentiate good prognosis (LA) and poor prognosis (LB) tumours in a cohort of predominantly ER+ early breast cancer patients enrolled in a randomised clinical trial of conservative surgery, post-operative whole breast radiotherapy and then randomised to an additional cavity boost or not. We previously described the clinical usefulness of a five biomarker panel (Millar *et al*, 2009b; ER, PR, HER2, CK 5/6 and EGFR) and have further defined luminal tumours by including Ki-67 and p53 status, the latter described in higher grade tumours, overexpressed more frequently within LB (Sorlie, 2004; Jacquemier *et al*, 2008; Hugh *et al*, 2009; Carey, 2010; Weigelt *et al*, 2010b) and as a predictor of endocrine resistance in some studies (Yamashita *et al*, 2006). These markers have easily available and well-characterised antibodies in current use, which can be immediately applied to clinical practise.

This study aimed to define the predictive value of a more refined luminal IHC biomarker signature in those patients who were ER+, with disease relapse and death from breast cancer as end-points.

## Materials and methods

### Study subjects

#### Training cohort

Cases were drawn from the St Vincent's Campus Outcome Cohort, which comprised 292 invasive ductal carcinomas treated between February 1992 and August 2002 at St Vincent's Hospital, Sydney, Australia. Ethics approval for use of tissue and clinicopathological data was granted by the Human Research Ethics Committee of St Vincent's Hospital, Sydney (Ref. SVH H94/080 and 00/036). A more detailed description of the clinicopathological characteristics of the cohort is published elsewhere (Millar *et al*, 2009a; López-Knowles *et al*, 2010). In summary, 40% of tumours were >20 mm, 45% were grade 3, 43% were lymph node positive, 68% were ER positive, 57% were PR positive and 18% were HER2 fluorescent *in situ* hybridisation (FISH) positive (HER2:CEP17 ratio >2.2). Median age was 54 years, and patients were treated with endocrine therapy (49%), chemotherapy (38%) or both (24%). Cases were prospectively followed up for a median of 64 months, and the outcome events measured were as follows: recurrence (local or distant; 25%), metastasis (23%) and breast cancer-specific death (18%). This cohort was used to identify differences in expression of several cell cycle and apoptotic markers, including Ki67 and p53 (CM McNeil *et al*, manuscript in preparation), between LA and B cancers using the following definitions: LA: ER+ and/or PR+ and HER2− and LB: ER+ and/or PR+ and HER2+. Using the median expression levels for Ki67 and p53 as the cutpoints (5% and 10%, respectively), we were able to demonstrate a significant difference in level of expression between LA and LB for these antigens (*P*=0.0158 and *P*=0.0061, respectively). Subsequently, we modified our definition of LA and LB to include Ki67 and p53 status as follows: ‘LA’: ER+ and/or PR+ and HER2−, Ki67 low and p53 negative and ‘LB’: ER+ and/or PR+ and/or HER2+ and/or Ki67 high and/or p53+. Kaplan–Meier analysis for breast cancer specific death showed a significant difference in outcome between these two groups of ER+ patients (*P*=0.0002) using this updated classifier (CM McNeil *et al*, manuscript in preparation).

#### Study validation cohort

**Figure 1:**
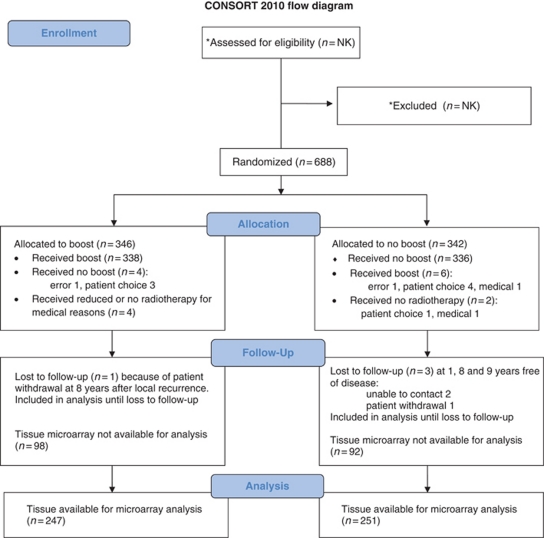
^*^The trial recruited from three main centres (St George, Wollongong and Liverpool Hospitals). Although the total number of patients assessed for eligibility and excluded for all centres is not known, this data are available for the main recruiting centres at St Geroge Hospital, which contributed the majority of patients in the trial, *n*=546 (number assessed, *n*=2046; excluded, *n*=1500: not meeting criteria, *n*=943; declined to partcipate, *n*=235; other reasons, *n*=322; patients randomised in the trials, *n*=536).

In this biomarker study, tissue was available from 498 patients (from a total of 688) with invasive breast cancer who were enrolled into a randomised clinical trial, which compared the benefit of the addition of a local cavity boost of radiotherapy to breast-conserving therapy (BCT; Clinical Trials Registry NCT00138814). The study was conducted at St George, Wollongong and Liverpool Hospitals, Sydney, New South Wales, Australia between 1996 and 2003 when the trial was closed to accrual. Follow-up for this analysis continued until September 2008. Clinicopathological details are summarised in Supplementary Table 1, and have been previously published in detail Millar *et al* (2009b). This study was approved by the Human Research Ethics Committee of the St George Hospital, Sydney, Australia (ref. no.: 96/84). The flow of patients through the trial is summarised in a CONSORT flow diagram (Figure 1). Patients were randomised using random blocking sequences set up before commencing of the study. Following patient consent, a person independent of the study both generated the sequence and assigned participants to interventions as below. This was an unblinded study.

All patients with invasive carcinoma received local excision and axillary sentinel node biopsy or axillary clearance. Adjuvant chemotherapy (AC or CMF) was given to 23.7% of patients and 44.9% received adjuvant endocrine therapy with tamoxifen. No patients received adjuvant trastuzumab. For patients subsequently classified as modified ‘LA’, 49.5% received endocrine therapy and 13.4% received chemotherapy, and those classified as modified ‘LB’ 55.7% received endocrine therapy and 25% received chemotherapy. Patients were randomised to whole breast radiotherapy of 50 Gy in 25 fractions or whole breast radiotherapy of 45 Gy in 25 fractions plus a tumour bed boost of 16 Gy in eight fractions. Supraclavicular fields were not added unless there were four or more nodes positive. In all, 17 patients had positive margins, 65 had clearance of <1 mm and a further 86 had <2 mm clearance, the remainder being well clear. HER2 status was unknown at the time of treatment.

### Study definitions

Patients were assessed at 6 weeks after radiation therapy, 6 monthly for 2 years, then annually thereafter with annual breast imaging. Follow-up time for this biomarker cohort was calculated from the date of the first surgical procedure to the date of the first event, as outlined below, or to the last known confirmed date of breast cancer disease-free status. Median follow-up time was 84 months (range 1–134 months). The primary end point was time to ipsilateral breast tumour recurrence (IBTR). This included any ipsilateral in-breast recurrence (invasive or non-invasive). The secondary end points were locoregional recurrence (LRR: IBTR, axilla, chest wall, internal mammary or supraclavicular fossa lymph nodes) and time to distant metastases and death.

### Tissue microarray (TMA) construction, IHC and FISH

TMAs were constructed from formalin-fixed paraffin-embedded tissue blocks, which were available from 498 invasive carcinomas, using 1 mm diameter punches with up to three cores sampled from each tumour. Antibodies used in IHC were Ki-67 (1 : 100, SP6 neomarkers), p53 (1:50, DO-7; Dako, Carpentaria, CA, USA), ER (1:100, 6F11; Dako), PR (1:200, PgR 636; Dako), CK 5/6 (1:80, MAB1602; Chemicon International, Temecula, USA), EGFR (1:100, H11; Dako).

All staining was performed using a Dako autostainer following antigen retrieval for all antibodies except for Ki-67, which was performed on a Leica (Wetzlar, Germany)/Bond Max system using ER2 (high pH antigen retrieval). All staining was centrally assessed by one breast Pathologist (EKAM). ER and PR were assessed as positive if a modified ‘H score’ (i.e., percentage × intensity) was >10. CK5/6 and EGFR were considered positive if staining of any intensity was present (i.e., >0). Tumours were considered HER2 positive only if they were HER2 amplified on FISH using a HER2: chromosome 17 ratio >2.2 as positive. p53 and Ki-67 were considered positive if there was >10% positive average nuclear staining of any intensity.

### Classification of intrinsic molecular phenotype

Patients were initially subtyped based on the status of their primary tumour as follows: ‘LA’: ER+ and/or PR+ and HER2−, and ‘LB’: ER+ and/or PR+ and HER2+ HER2 enriched: ER− and PR− and HER2+, and basal: ER−, PR−, HER2−, CK 5/6+ and/or EGFR +, unclassified (negative for all five markers). Subsequently they were re-classified as modified ‘LA’: ER+ and/or PR+ and Ki-67 low, p53−, HER2− modified ‘LB’: ER+ and/or PR+ and/or Ki-67 high and/or p53+ and/or HER2+ HER2 enriched: ER− and PR− and HER2+, and basal: ER−, PR−, HER2−, CK 5/6+ and/or EGFR+, unclassified (negative for all five markers).

### Statistical analyses

Kaplan–Meier analyses for IBTR, LRR, distant disease-free survival and breast cancer-specific death were estimated for each subtype and compared using the log-rank test. We used Cox proportional hazards univariate analysis to analyse the association between prognostic variables and molecular subtype with IBTR, LRR, metastases and breast cancer-specific death. Multivariate analysis (MVA) was used to construct models identifying those variables which were independently prognostic. Subsequently, step-wise removal of variables was used until resolution. Analyses were performed using Statview 5.0 (Abacus systems, Berkeley, CA, USA) and STATA 10.0 (StataCorp LP, College Station, TX, USA). The ANOVA was used to assess differences in expression of target antigens as continuous variables between intrinsic subtypes.

## Results

### Assessment of Ki67 and p53 expression between LA and B tumours

Having identified differences in Ki67 and p53 in ER+ tumours in our training cohort, we then assessed the difference between LA and B tumours in expression level of these two antigens in our validation cohort (*n*=498). Within LB tumours in this cohort, we observed significantly higher levels of Ki-67 and p53 expression (*P*=0.0008 and 0.0048, respectively). The median average value for both Ki67 and p53 within the validation cohort was 10%. Subsequently, we modified our working definition further for good prognosis modified ‘LA’ as ER+ and/or PR+ and HER2−, Ki67 low and p53− and poor prognosis modified ‘LB’ as ER+ and/or PR+ and/or HER2+ and/or Ki67 high and/or p53+.

### Five-year survival rates, univariate analysis of LA and B tumours for IBTR, LRR, distant metastasis-free survival (DMFS) and breast cancer-specific survival (BCSS)

Using these updated definitions, 321 tumours (64.5%) were classified as LA and 96 as LB (19.3%). Thus, 73 previously LA tumours were re-classified as LB (previously only 23 tumours were LB), that is, 4.2-fold increase (4.6–19.3%) with LB now comprising 23% of all ER+ tumours. We then examined the relative contribution of p53 and Ki67 to the updated classification of the 96 LB tumours: 57 of 96 (59.4%) were p53−/Ki67+, 19 (19.7%) were p53+/Ki67−, 12 (12.5%) were p53+/Ki67+ and 8 were p53−/Ki67− (HER2+).

As previously described, no benefit of a tumour bed boost was observed in this group of patients (Millar *et al*, 2009b). At a median follow-up period of 84 months, the 5-year survival rates for modified LA and modified LB, respectively, using the updated classifier were IBTR 99.3, 96.6% LRR 99.7, 93.4% DMFS 97, 87% and BCSS 99.7, 92.5%. Comparative analyses of the clinicopathological features, crude event rates and univariate analyses of LA and LB groups between the differing definitions are presented in Tables 1 and 2. Univariate Cox proportional hazards were calculated for each measure of outcome for Ki67 and p53 and the modified LA and LB subtypes, which are presented with crude event rates in Table 3. Further crude event rates for modified LA and LB for lymph node negative, lymph node positive and lymphatic vascular invasion are presented in Supplementary Table 2. As expected, the updated classification resulted in increased numbers of events for all outcomes for LB and a reduction for LA. This is mirrored in LB by increases in LVI and LN+ status, with recurrence rates and death rates two to three times that of LA. Univariate analyses showed that modified LA is a significant predictor for all measures of outcome including IBTR (hazard ratio (HR) 0.314, 95% CI 0.136–0.726, *P*=0.007) where it previously was close to but not statistically significant (*P*=0.051). Modified LB predicted DMFS and BCSS (*P*=0.005 and 0.003, respectively) and approached significance for IBTR and LRR (*P*=0.07 and 0.052, respectively) where previously it was not significant for any outcome measure. Ki67 predicted outcome for all measures (BCSS: HR 4.98, 95% CI 2.530–9.694, *P*<0.0001). p53+ predicted DMFS and BCSS (HR 3.523, 95% CI 1.731–7.168, *P*=0.0005) but not IBTR or LRR.

### Kaplan–Meier analysis of intrinsic subtype

**Figure 2:**
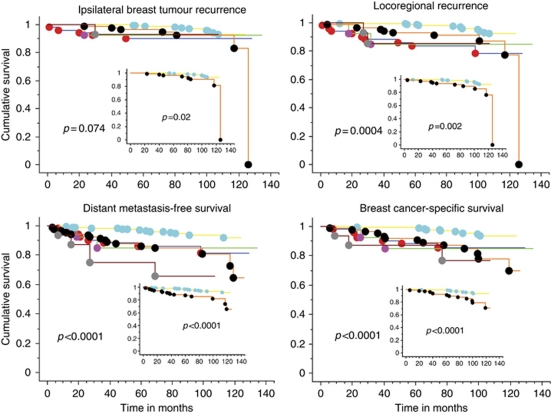
Kaplan–Meier estimates for ipsilateral breast tumour recurrence, locoregional recurrence, distant metastasis-free survival and breast cancer-specific survival for all intrinsic subtypes and for luminal A *vs* luminal B (inserts). Luminal A 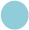 *n*=321, luminal B 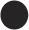 *n*=96, basal 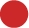 *n*=52, HER2 enriched 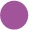 *n*=13, unclassified 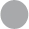 *n*=16.

Kaplan–Meier analysis (log-rank test) comparing modified LA and LB alone was significant for all measures of outcome IBTR *P*=0.02, LRR *P*=0.002, DMFS and BCSS both *P*<0.0001 (Figure 2 inserts). This classifier also showed improvement in the degree of statistical significance between all molecular subtypes compared with the previously reported five biomarker panel, which was observed for LRR *P*=0.0004 (previously 0.012), DMFS *P*<0.0001 (previously 0.0035) and BCSS *P*=0.0001 (previously 0.048) but not for IBTR (*P*=0.074, previously 0.346, Figure 2). Although LA had an excellent prognosis, LB had adverse survival, similar to basal, HER2-enriched and unclassified subtypes.

### MVA for IBTR, LRR, DMFS and BCSS

We then constructed multivariable models of clinicopathological features and intrinsic subtype to assess predictive value and compare HRs between intrinsic subtypes, using modified LA as a reference group.

### Ispilateral breast tumour recurrence

Only margin status (HR 3.158, 95% CI 1.067–9.348, *P*=0.378) and grade (HR 3.13, 95% CI 1.4–7.012, *P*=0.0055) independently predicted recurrence with no other prognostic variable or intrinsic subtype reaching statistical significance.

### Locoregional recurrence

Luminal B (HR 3.612, 95% CI 1.555–8.340, *P*=0.003), basal, unclassified and extensive intraduct carcinoma positive were independent predictors of outcome in the final resolved model (Table 4).

### DMFS and BCSS

Luminal B was an independent predictor of adverse outcome for both metastases and breast cancer-specific death in the final resolved models (LB DMFS: HR 3.023, 95% CI 1.501–6.089, *P*=0.002; BCSS: HR 3.617, 95% CI 1.629–8.031, *P*=0.002), along with LVI, LN positivity, basal and unclassified (Table 4).

## Discussion

Oestrogen receptor-positive early breast cancer is the commonest form of the disease and tailoring treatment to individual patients is a priority. It is important to identify ER+ patients with a good prognosis who will receive most benefit from endocrine therapy and receive little or no benefit from chemotherapy, and, therefore, avoid any toxicity. In addition, it is also beneficial to identify patients who will have little or no benefit from endocrine therapy. GEP studies have consistently identified at least two groups of ER+ tumours; the less favourable LB group being characterised by higher histological grade and higher expression of proliferation and HER2-related genes, such as *MKI67*, *MYBBL2*, *CCNB1*, *HER2* and *GRB7*, and lower levels of *ER*-related genes. Although there is some consistency in the recognition of these differing subgroups between GEP studies, there is some doubt as to the stability of the classifiers used by different single sample predictors (Weigelt *et al*, 2010b) and most assays are not yet ready for routine clinical use (De Ronde *et al*, 2010). As a result, a simple and relatively cheap test using IHC surrogates would be easier to transfer into clinical practise. Various combinations of markers have been assessed to develop a robust IHC panel for routine pathology reporting, most recently adding Ki67 to ER, PR, HER2 to better assess proliferative luminal tumours (Cheang *et al*, 2009; Hugh *et al*, 2009). Assessing ER+ tumours with surrogates for molecular grade may strengthen patient selection as histological grade can be compromised in some specimens because of sub-optimal fixation.

Using an independent discovery cohort of 292 patients, we identified a significant difference in expression in Ki-67 and p53 within ER+ cancers, which was associated with differences in clinical outcomes (breast-cancer specific death; CM McNeil *et al*, manuscript in preparation). These findings were subsequently validated in a detailed analysis of 498 early breast cancer patients, in which we compared good and poor prognosis ‘LA’ and ‘LB’ IHC signatures, which included Ki-67 and p53 in addition to ER, PR and HER2. This updated definition provided superior predictive power and better discrimination between the two groups of luminal tumours for all measures of outcome. In all, 73 previously LA tumours were reclassified as LB, increasing the size of the ‘LB’ group by >four-fold from 4.6 to 19.7% of the cohort, better reflecting GEP estimates of the size of the LB population. Using this definition, ‘LB’ was an independent predictor of poor prognosis in MVA for LRR, DMFS and BCSS but not for IBTR for the whole cohort. As well as demonstrating its superior predictive power over the most frequently used classifier or ER, PR, HER2 alone, we also performed additional analyses to make a comparison with ER+ breast cancer classified by hormone receptor (HR) status alone (data not shown). Some studies have shown a significant difference in outcome between double-positive (i.e., ER+ PR+) and single-receptor positive HR status (i.e., ER+ PR− or ER− PR+, Rakha *et al*, 2007). This latter group may correspond to the LB subtype (Rakha *et al*, 2009). Our further analyses of these subgroups demonstrated that HR status alone was inferior to our updated five biomarker classifier: in univariate analysis good prognosis double-positive status (ER+ PR+) was only statistically predictive for distant metastases and death (not IBTR or LRR) and single-positive status (i.e., poor prognosis ‘LB’) was not predictive for any measure of outcome in univariate analysis.

Our updated classification of ER+ disease also improves the statistical significance in survival between all intrinsic subtypes, where the adverse survival and HR of our poor prognosis ‘LB’ group is three times that of ‘LA’ and closer to that of HER2-enriched and basal subtypes. One limitation of this study is that recurrence rates may be over estimated for LB, as the prognosis of HER2-positive LB tumours (24% of all LB tumours) would currently be modified by the benefits of Herceptin treatment (which was not used in this study) and an underestimate for LA, as only 44.9% of patients received adjuvant tamoxifen. An additional limitation of this study is the difference in cut points used for Ki67 positivity where the training cohort median was 5% and the validation cohort median was 10%. Although we have identified good and poor prognostic groups with our signature, the relatively wide confidence intervals, which reflect the small numbers of events, strongly suggests the importance of further independent validation. Further analyses in a larger data set with a greater number of events may provide narrower confidence intervals, which along with assessment of the hazard ratio will determine the likely clinical significance derived from this panel of markers.

These findings suggest a potential role for this biomarker panel in better defining groups of ER+ cancer of low and high molecular grade, allowing better selection of patients for endocrine therapy alone or with AC. Although Ki67 alone identifies approximately 60% of LB tumours, p53 adds a further 20% of cases, 12% are positive for both markers, 8% are negative for both but HER2 positive. This study builds upon previous work (Cheang *et al*, 2009) using a cut point for optimal determination of ‘high’ Ki-67 proliferation rate at 14% through correlation with the PAM50 classifier using RT–PCR. They identified a LB population, which was 42% of the cohort (includes their LB and luminal HER2 cases). Although the cut point of 14% correlates with GEP estimates it may, in practical terms, be difficult to discern by IHC. Ki67 has long been analysed in breast cancer cohorts with varied results in terms of its predictive value. A recent review has recommended its inclusion as a routine biomarker in breast cancer (Yerushalmi *et al*, 2010), but its application as a stand alone biomarker has been debated (Stuart-Harris *et al*, 2008). Therefore, its inclusion in a panel to help define molecular grade and better subtype ‘LA’ and ‘LB’ cancers is independently prognostic and valuable. However, its role as a predictive marker appears less certain. A pre- and post-biopsy analysis of endocrine treated breast cancer has demonstrated that only the post-treatment tumour Ki67 (at 2 weeks) was predictive of response to endocrine therapy, whereas baseline Ki67 was not (Dowsett *et al*, 2007). High Ki67 status in BIG 1–98 suggested a potential benefit in selecting letrozole over tamoxifen in post-menopausal patients (Viale *et al*, 2008). Most recently a significant study identified that the prognostic information provided by ‘IHC4’ (ER, PR, HER2 and Ki-67) was at least equivalent to Oncotype Dx (Cuzick *et al*, 2009) and highlights the relevance of these readily available routine pathology markers in the clinical management of breast cancer.

p53 overexpression in breast cancer assessed by IHC is, rather over simplistically, assumed to act as a surrogate for TP53 mutations and is associated with higher tumour grade and responsiveness to radiotherapy, chemotherapy and endocrine therapy (Thompson and Lane, 2010). Although the p53 pathway is undoubtedly highly complex, its assessment by IHC does appear to provide meaningful information. p53 mutations are more frequent in the LB group compared with LA (Weigelt *et al*, 2010a), being described in 71% of LB tumours but only 16% of LA (Sorlie, 2004). p53 currently features as one of five antibodies in the Mammostrat (Clarient, Inc., Aliso Viejo, CA, USA) IHC test shown to be of predictive value in ER+, tamoxifen-treated early breast cancer (Ring *et al*, 2006; Bartlett *et al*, 2010). Mammostrat uses a five IHC panel (p53, HTF9C, CEACAM5, NDRG1, SLC7A5) with an algorithm that is independent of ER and PR status to identify low-, medium- and high-risk groups. The initial published study (Ring *et al*, 2006) demonstrated HRs of 1.8 and 2.3 (training and validation cohorts, respectively) for high risk compared with the low and medium risks for disease recurrence. Elevated expression of p53 was observed by IHC in our cohorts and appeared to be a useful classifier and was included in the updated definition of poor prognosis ‘LB’ cancer.

Although the number of events was small, additional exploratory multivariate analyses for patients treated with tamoxifen alone (*n*=169, 10 events) showed that the poor prognosis ‘LB’ definition retained independent prognostic significance in the final resolved model for breast cancer specific death (HR 5.361, 95% CI 1.418–20.25, *P*=0.013). This finding suggests that ‘LB’ has five times the risk of death compared with ‘LA’ in patients treated with endocrine therapy. The predictive value of this classification would however require further testing within the setting of a randomised trial of endocrine therapy.

Our updated definition of ER+ cancer translates into an IBTR-free survival at 5 years of 99.3% for LA and 96.6% LB, LRR-free survival 99.7 and 93.4%. A similar recent study using ER, PR and Ki67 in the definition for LA and LB found local recurrence-free rates at 10 years of 92% for LA and 90% for LB (Voduc *et al*, 2010). Importantly, our findings further support the observations of this group, who found that LB was associated with increased risk of LRR. These results highlight the role of proliferation and grade, mirrored by the Oncotype Dx assay (Mamounas *et al*, 2005), as a predictor of locoregional recurrence, and may help further refine patient selection regarding therapy for optimal locoregional control. A subsequent study analysed patterns of metastases and found both LA and LB had a predilection for bone as a metastatic site and found that LB had a distant relapse rate similar to basal tumours at 15 years (Kennecke *et al*, 2010). In summary, this study suggests that good and poor prognosis ER+ breast cancers can be reliably and easily discriminated using Ki67 and p53 in addition to ER, PR and HER2 in routine pathology IHC. This definition greatly enhances the detection of poor prognosis ER+ ‘LB’ breast cancers, with an outcome closer to that of basal and HER2-enriched tumours. This approach may help more reliably define groups of ER+ patients with an excellent prognosis and identify those at risk of early relapse who may benefit from more frequent follow-up and early intervention with alternative therapies and/or chemotherapy. Further, larger studies in randomised clinical trials of endocrine therapy are required to assess the clinical utility of this classification and its value as a predictor of therapeutic responsiveness.

## Figures and Tables

**Table 1:** Patient tumour characteristics and event rates classified by luminal phenotype

	**Whole cohort, *n*=498 (%)**	**Luminal A, *n*=394 (79.1%)**	**Luminal B, *n*=23 (4.6%)**	**Modified luminal A, *n*=321(64.5%)**	**Modified luminal B, *n*=96 (19.3%)**
*Patient tumour characteristics*					
Size <20 mm	357 (70.3)	289 (73.4)	17 (73.9)	242 (75.4)	64 (66.7)
LVI+	79 (15.8)	62 (15.7)	4 (17.4)	43 (13.4)	23 (23.9)
LN+	146 (29.0)	117 (29.6)	5 (21.7)	86 (26.7)	36 (37.5)
Grade 3	145 (29.1)	65 (16.5)	16 (69.5)	26 (8.1)	55 (57.3)
EIC+	45 (9.0)	29 (7.4)	5 (21.7)	23 (7.2)	11 (11.5)
Median Age	61	62	57	62	61
					
*Events*					
Median follow-up	84	83.5	71	84	78
IBTR	24 (4.8)	15 (3.8)	2 (8.7)	9 (2.8)	8 (8.3)
LRR	35 (7.0)	20 (5.1)	2 (8.7)	11 (3.4)	11 (11.5)
DMFS	47 (9.4)	30 (7.6)	2 (8.7)	16 (4.9)	16 (16.7)
BCSS	37 (7.4)	23 (5.8)	2 (8.7)	11 (3.4)	14 (14.6)

Abbreviations: BCSS=breast cancer-specific survival; DMFS=distant metastasis-free survival; EIC=extensive intraduct carcinoma; ER+ =oestrogen receptor positive; IBTR=ipsilateral breast tumour recurrence; LN=lymph node; LRR=locoregional recurrence; LVI=lymphatic/vascular invasion; PR+ =progesterone receptor positive.

Luminal A: ER+ and/or PR+, HER2− Luminal B: ER+ and/or PR+, HER2+ modified luminal A: ER+ and/or PR+, Ki67 low and p53− and HER2− modified luminal B: ER+ and/or PR+ and/or Ki67 high and/or p53+ and/or HER2+.

**Table 2:** Comparative 5 and 10 year event rates for luminal A and B

	**IBTR**		**LRR**		**DM**		**BCSD**	
	**5 Year (%)**	**10 Year (%)**	**5 Year (%)**	**10 Year (%)**	**5 Year (%)**	**10 Year (%)**	**5 Year (%)**	**10 year (%)**
Whole cohort (*n*=498)	12/498 (2.4)	23/498 (4.6)	21/498 (4.2)	35/498 (6.8)	34/498 (6.8)	47/498 (9.4)	18/498 (3.6)	37/498 (7.4)
	12/24 (50)	23/24 (95.8)	21/35 (60)	35/35 (100)	34/47 (72.3)	47/47 (100)	18/37 (48.6)	37/37 (100)
Luminal A (*n*=394)	4/394 (1)	14/394 (3.6)	8/394 (2)	19/394 (4.8)	19/394 (4.8)	30/394 (7.6)	7/394 (1.8)	23/394 (5.8)
	4/15 (26.6)	14/15 (93.3)	8/20 (40)	19/20 (95)	19/30 (63.3)	30/30 (100)	7/23 (30.4)	23/23 (100)
Luminal B (*n*=23)	1/23 (4.3)	2/23 (8.7)	1/23 (4.3)	2/23 (8.6)	2/23 (8.6)	2/23 (8.6)	1/23 (4.3)	2/23 (8.6)
	1/2 (50)	2/2 (100)	1/2 (50)	2/2 (100)	2/2 (100)	2/2 (100)	1/2 (50)	2/2 (100)
Modified luminal A (*n*=321)	2/321 (0.6)	9/321 (2.8)	3/321 (0.9)	11/321 (3.4)	9/321 (2.8)	16/321 (4.9)	1/321 (0.3)	11/321 (3.4)
	2/9 (22.2)	9/9 (100)	3/11 (27)	11/11 (100)	9/16 (56.3)	16/16 (100)	1/11 (9.1)	11/11 (100)
Modified luminal B (*n*=96)	3/96 (3.1)	7/96 (7.3)	6/96 (6.3)	10/91 (10.9)	12/96 (12.5)	16/96 (16.7)	7/96 (7.3)	14/96 (14.6)
	3/8 (37.5)	7/8 (87.5)	6/11 (54.5)	10/11 (90.1)	12/16 (75)	16/16 (100)	7/14 (50)	14/14 (100)

Abbreviations: BCSD=breast cancer-specific death; DM=distant metastasis; ER+ =oestrogen receptor positive; IBTR=ipsilateral breast tumour recurrence; LRR=locoregional recurrence; PR+ =progesterone receptor positive.

Modified luminal A: ER+ and/or PR+, Ki-67 low, p53−, HER2− modified luminal B: ER+ and/or PR+ and/or Ki-67 high and/or p53+ and/or HER2+. In the top row of each box, the denominator is the total number of patients within that patient group or subtype; in the bottom row of each box, the denominator is the total number of events for each group or subtype.

**Table 3:** Univariate crude rates and hazard ratio (Cox) for biomarkers and luminal phenotype

	**IBTR (*n*=24)**			**LRR (*n*=35)**			**DDFS (*n*=47)**			**BCSS (*n*=37)**		
	**CR**	**HR (95% CI)**	***P***	**CR**	**HR (95% CI)**	***P***	**CR**	**HR (95% CI)**	***P***	**CR**	**HR (95% CI)**	***P***
Ki67 high	12/129	3.126 (1.390–7.029)	**0.0008**	19/129	3.759 (1.923–7.340)	**0.0001**	24/129	3.436 (1.926–6.130)	**<0.0001**	22/129	4.948 (2.530–9.674)	**<0.0001**
p53+	3/57	1.067 (0.315–3.629)	0.916	5/57	1.290 (0.497–3.350)	0.601	11/57	2.566 (1.303–5.056)	**0.006**	11/57	3.523 (1.731–7.168)	**0.0005**
LA	15/394	0.433 (0.186–1.005)	**0.051**	20/394	0.333 (0.169–0.655)	**0.002**	30/394	0.446 (0.246–0.810)	**0.008**	23/394	0.414 (0.213–0.816)	**0.009**
LB	2/23	2.132 (0.500–9.098)	0.307	2/23	1.365 (0.327–5.697)	0.669	2/23	0.963 (0.233–3.971)	0.958	2/23	1.258 (0.302–5.234)	0.753
Modified LA	9/321	0.314 (0.136–0.726)	**0.007**	11/321	0.233 (0.113–0.478)	**<0.0001**	16/321	0.263 (0.144–0.481)	**0.0001**	11/321	0.218 (0.108–0.441)	**<0.0001**
Modified LB	8/96	2.217 (0.0.945–5.200)	0.07	11/96	2.036 (0.995–4.167)	0.052	16/96	2.351 (1.285–4.300)	**0.005**	14/96	2.733 (1.406–5.314)	**0.003**

Abbreviations: CI=confidence interval; CR=crude rate; DMFS=distant metastasis-free survival; ER+ =oestrogen receptor positive; HR=hazard ratio; IBTR=ipsilateral breast tumour recurrence; LA=luminal A; LB=luminal B; LRR=locoregional recurrence; PR+ =progesterone receptor positive.

LA: ER+ and/or PR+ and HER2− LB: B ER+ and/or PR+ and HER2+ modified LA: ER+ and/or PR+, Ki-67 low, p53−, HER2− modified LB: ER+ and/or PR+ and/or Ki-67 high and/or p53+ and/or HER2+.

Bold typescript indicates statistical significance.

**Table 4:** Cox proportional hazards multivariate models

**Variable**	**HR**	**95% CI**	***P***
*Locoregional recurrence*			
Grade 3	1.938	0.823–4.568	0.130
Size>20 mm	0.861	0.408–1.817	0.694
LN+	2.188	1.054–4.542	0.036
LVI	1.286	0.546–3.026	0.564
EIC+	3.136	1.328–7.405	0.009
			
*Subtype*			
Modified LA (reference)	1.0		
Modified LB	2.483	0.982–6.281	0.055
Basal	3.939	1.281–12.114	0.017
HER2	1.931	0.382–9.754	0.426
Unclassified	4.471	0.926–21.59	0.062
			
*Resolved model*			
EIC+	2.476	1.070–5.730	**0.034**
Modified LB	3.612	1.555–8.340	**0.003**
Basal	5.541	2.279–13.47	**<0.001**
HER2	3.549	0.764–16.51	0.106
Unclassified	4.913	1.077–22.42	**0.040**
			
*Distant metastasis free survival*			
Grade 3	1.100	0.529–2.287	0.879
Size>20 mm	1.372	0.742–2.540	0.313
LN+	3.822	2.036–7.175	<0.001
LVI	1.832	0.960–3.499	0.067
			
*Subtype*			
Modified LA (reference)	1.0		
Modified LB	2.872	1.326–6.222	0.007
Basal	3.273	1.139–9.396	0.028
HER2	1.825	0.386–8.639	0.448
Unclassified	9.902	3.269–29.99	<0.001
			
*Resolved model*			
LN+	4.013	2.154–7.477	**<0.001**
LVI	2.011	1.075–3.764	**0.029**
Modified LB	3.023	1.501–6.089	**0.002**
Basal	3.902	1.657–9.191	**0.002**
HER2	2.064	0.472–9.026	0.336
Unclassified	10.87	3.882–30.461	**<0.001**
			
*Breast cancer specific death*			
Grade 3	1.307	0.570–2.997	0.527
Size>20 mm	1.879	0.927–3.807	0.080
LN+	4.535	2.153–9.553	<0.001
LVI	2.085	1.030–4.223	0.041
			
*Subtype*			
Modified LA (ref)	1.0		
Modified LB	3.084	1.280–7.431	0.012
Basal	3.780	1.155–12.37	0.028
HER2	2.095	0.412–10.65	0.373
Unclassified	8.167	1.997–33.40	0.003
			
*Resolved model*			
LN+	4.906	2.353–10.22	**<0.001**
LVI	2.518	1.267–5.004	**0.008**
Modified LB	3.617	1.629–8.031	**0.002**
Basal	5.715	2.173–15.03	**<0.001**
HER2	2.907	0.641–13.17	0.166
Unclassified	10.37	2.801–38.42	**<0.001**

Abbreviations: CI=confidence interval; EIC+ =extensive intraduct component of DCIS=ductal carcinoma *in situ*; HR=hazard ratio; LA=luminal A; LB=luminal B; LN=lymph node; LVI=lymphatic vascular invasion.

Bold typescript indicates statistical significance.
