# Supplementary material for: Prediction of outcome of early ER+ breast cancer is improved using a biomarker panel, which includes Ki-67 and p53
Source: Br J Cancer. 2011 Jun 28;105(2):272–80. doi: 10.1038/bjc.2011.228 (PMC3142808; doi:10.1038/bjc.2011.228)
Supplement: Supplementary Table 1 [file bjc2011228x1.doc]

**­­­­­­­­­­­­­­­­­­­­Supplementary Table 1**. Patient baseline characteristics, treatments and outcomes.

Characteristic No of patients (%) Median Range

Length of follow-up (months) 498 84 1-134

Age (years) 61 24-84

Tumor size (mm) 16 1-60

T1a (1- 5) 4 (0.8)

T1b (6 -10) 77(16.3)

T1c (11-20) 270(54.2)

T2 (21-50) 136(27.3)

T3 (>50) 1(0.2)

Tumor Grade

1 167 (33.5)

2 185 (37.1)

3 145 (29.1)

Lymph node metastases 146(29.3)

N0 339(69.9)

N1 (1-3) 128 (25.7)

N2 (4-10) 17(3.5)

N3 (>10) 2 (0.4)

LN unsampled 12 (2.4)

ER+ 393 (78.9)

PR+ 334( 68.3)

HER-2 amplified (FISH) 36 (7.2)

Luminal A 394 (79.1) Modified Luminal A 321 (64.5%)

Luminal B 23 (4.6) Modified Luminal B 96 (19.3%)

Basal-like 52 (10.4)

HER-2 13 (2.6)

Unclassified 16 (3.2)

Triple negative 68 (13.6)

Margin + 17 (3.4)

Cavity boost positive 247 (49.5)

Cavity boost negative 251 (50.5)

Endocrine therapy 223 (44.7)

Chemotherapy 117 (23.4)

Endocrine & chemotherapy 48 (9.6)

Patients with IBTR 24 (4.8)

Patients with LRR 35 (7)

Patients with distant metastases 47 (9.4)

Breast cancer specific deaths 37 (7.4)

5 year IBTR free survival 97.4%

5 year LRR free survival 95.6%

5 year DDFS 92.9%

5 year breast cancer-specific survival 96.3%
