# Supplementary material for: Prediction of outcome of early ER+ breast cancer is improved using a biomarker panel, which includes Ki-67 and p53
Source: Br J Cancer. 2011 Jun 28;105(2):272–80. doi: 10.1038/bjc.2011.228 (PMC3142808; doi:10.1038/bjc.2011.228)
Supplement: Supplementary Table 2 [file bjc2011228x2.doc]

Supplementary Table 2 . Crude event rates for Luminal A and Luminal B according to lymph node and lymphatic vascular status.

IBTR LRR Distant Metastases Breast Cancer Death

n 5yr (%) 10 yr (%) 5yr (%) 10yr (%) 5yr (%) 10yr (%) 5yr (%) 10yr (%)

LN- LA 223 1/223 (0.4) 6/223 (2.7) 2/223 (0.9) 8/223 (3.6) 2/223 (2.7) 6/223 (2.7)) 1/223 (0.4) 4/223 (1.8)

LB 60 1/60 (1.7) 2/60 (3.3) 2/60 (3.3) 4/60 (6.7) 4/60 (6.7) 5/60 (8.3) 2/60 (3.3) 3/60 (5)

LN + LA 86 1/86 (1.2) 3/86 (3.5) 1/86 (1.2) 3/86 (3.5) 7/86 (8.1) 10/86 (11.6) 0/86 (0) 7/86 (8.1)

LB 36 2/36 (5.5) 5/36 (13.9) 4/36 (11.1) 6/36 (16.7) 8/36 (22.2) 11/36 (30.5) 6/36 (16.7) 11/36 (30.5)

LVI + LA 43 0/43 (0) 0/43 (0) 0/43 (0) 0/43 (0) 3/43 (6.9) 4/43 (9.3) 0/43 (0) 3/43 (6.9)

LB 23 1/23 (4) 3/23 (13) 4/23 (17.4) 5/23 (21.7) 5/23 (21.7) 8/23 (34.7) 4/23 (17.4) 8/23 (34.7)

IBTR: ipsilateral breast tumor recurrence, LRR: locoregional recurrence, LN-: lymph node negative, LN+: lymph node positive, LVI+: lymphatic vascular invasion positive, LA : luminal A ER+ and/or PR+, HER2-, KI67 low, p53-; LB: luminal B: ER+ and/or PR+ and HER2+ and/or Ki67 high and/or p53+. LN status was not assessed in 12 LA patients.
